# Defining the structure–function relationship of specific lesions in early and advanced age-related macular degeneration

**DOI:** 10.1038/s41598-024-54619-3

**Published:** 2024-04-16

**Authors:** Ting Fang Tan, Chun Lin Yap, Claire L. Peterson, Damon Wong, Tien Yin Wong, Chui Ming Gemmy Cheung, Leopold Schmetterer, Anna Cheng Sim Tan

**Affiliations:** 1 Singapore National Eye Centre, Singapore General Hospital, 11 Third Hospital Avenue, Singapore, 119228 Singapore; 2 Singapore Eye Research Institute, Singapore, Singapore; 3 Tsinghua Medicine, Tsinghua University, Beijing, China; 4 Ophthalmology and Visual Sciences Academic Clinical Program, Duke-National University of Singapore Medical School, Singapore, Singapore; 5 School of Chemistry, Chemical Engineering and Biotechnology, Nanyang Technological University, Singapore, Singapore; 6 Department of Clinical Pharmacology, Medical University Vienna, Vienna, Austria; 7 Center for Medical Physics and Biomedical Engineering, Medical University Vienna, Vienna, Austria; 8 Institute of Molecular and Clinical Ophthalmology, Basel, Switzerland

**Keywords:** Age related macular degeneration, Imaging, Microperimetry, Multimodal, Visual function, Retinal diseases, Medical imaging

## Abstract

The objective of this study is to define structure–function relationships of pathological lesions related to age-related macular degeneration (AMD) using microperimetry and multimodal retinal imaging. We conducted a cross-sectional study of 87 patients with AMD (30 eyes with early and intermediate AMD and 110 eyes with advanced AMD), compared to 33 normal controls (66 eyes) recruited from a single tertiary center. All participants had enface and cross-sectional optical coherence tomography (Heidelberg HRA-2), OCT angiography, color and infra-red (IR) fundus and microperimetry (MP) (Nidek MP-3) performed. Multimodal images were graded for specific AMD pathological lesions. A custom marking tool was used to demarcate lesion boundaries on corresponding enface IR images, and subsequently superimposed onto MP color fundus photographs with retinal sensitivity points (RSP). The resulting overlay was used to correlate pathological structural changes to zonal functional changes. Mean age of patients with early/intermediate AMD, advanced AMD and controls were 73(SD = 8.2), 70.8(SD = 8), and 65.4(SD = 7.7) years respectively. Mean retinal sensitivity (MRS) of both early/intermediate (23.1 dB; SD = 5.5) and advanced AMD (18.1 dB; SD = 7.8) eyes were significantly worse than controls (27.8 dB, SD = 4.3) (p < 0.01). Advanced AMD eyes had significantly more unstable fixation (70%; SD = 63.6), larger mean fixation area (3.9 mm^2^; SD = 3.0), and focal fixation point further away from the fovea (0.7 mm; SD = 0.8), than controls (29%; SD = 43.9; 2.6 mm^2^; SD = 1.9; 0.4 mm; SD = 0.3) (p ≤ 0.01). Notably, 22 fellow eyes of AMD eyes (25.7 dB; SD = 3.0), with no AMD lesions, still had lower MRS than controls (p = 0.04). For specific AMD-related lesions, end-stage changes such as fibrosis (5.5 dB, SD = 5.4 dB) and atrophy (6.2 dB, SD = 7.0 dB) had the lowest MRS; while drusen and pigment epithelial detachment (17.7 dB, SD = 8.0 dB) had the highest MRS. Peri-lesional areas (20.2 dB, SD = 7.6 dB) and surrounding structurally normal areas (22.2 dB, SD = 6.9 dB) of the retina with no AMD lesions still had lower MRS compared to controls (27.8 dB, SD = 4.3 dB) (p < 0.01). Our detailed topographic structure–function correlation identified specific AMD pathological changes associated with a poorer visual function. This can provide an added value to the assessment of visual function to optimize treatment outcomes to existing and potentially future novel therapies.

## Introduction

Rapidly aging populations will lead to increasing rates of age-related macular degeneration (AMD), particularly in Asia^1,2^. AMD progression can potentially lead to decline in visual function (VF), worsening quality of life and independence with activities of daily living^3–5^. In AMD, structure–function correlation is important in understanding the impact of disease progression and treatment response on VF^4,5^. Visual acuity (VA) is the current standard for VF assessment, in both clinical practice and clinical trials. However, previous studies have highlighted the limitations of using only VA, which is poorly correlated to disease progression and impact on patients' VF^6,7^. Hence additional measurements of VF may better reflect structure–function correlation^6,7^. Among these, microperimetry (MP) has been demonstrated to be more sensitive in detecting subtle changes in visual decline^8–10^.

Multimodal non-invasive structural imaging, including color fundus photograph (CFP), spectral-domain optical coherence tomography (OCT), OCT angiography (OCTA) and infra-red (IR) fundus images, is the standard of care for diagnosis and monitoring of treatment outcomes in AMD^11,12^. OCT in particular has revolutionized the understanding of AMD through depth-resolved imaging in identifying various unique pathological AMD-related lesions^13–15^. The recent addition of OCTA further enabled visualization of retinal vasculature to identify neovascularization (NV), which may not be evident on OCT and previously only appreciable using invasive techniques like fluorescein and indocyanine green angiography^14,15^.

Existing studies have demonstrated that MP enabled direct function-structure correlation to specific AMD-related lesions identified on enface imaging^16–18^. However, previous studies mainly used single imaging modality (OCT). Other studies correlated MP retinal sensitivity (RS) measurements to structural lesions, via superimposing the entire macular region or using sectors such as the Early Treatment of Diabetic Retinopathy Study (ETDRS) grid^16,17^.

Hence, our study aims to perform a detailed topographic pointwise correlation of VF on MP, with specific AMD-related lesions identified on multimodal imaging (CFP, OCT and OCTA). We hypothesize that lesions such as drusen and pigment epithelial detachment (PED), subretinal fluid (SRF) and intraretinal fluid (IRF) will have better topographic RS than late chronic lesions such as fibrosis and atrophy.

## Methods

### Study population

This was a cross-sectional observational cohort study that involved 87 participants with AMD and 33 normal controls without disease. Participants were recruited from the Singapore National Eye Centre between November 2017 and December 2021. Written and informed consent was obtained from all participants. Participants with missing data were removed by listwise deletion. This study was in accordance with the Declaration of Helsinki and approval was obtained from the Centralised Institutional Review Board of the Singapore Eye Research Institute.

The inclusion criteria for AMD patients included: (a) > 40 years of age, (b) Presence of at least 1 eye with AMD, (c) AMD lesion that does not extend past the superior and inferior vascular arcades; and for normal controls: (a) > 40 years of age, (b) No lesions associated with AMD, i.e. no drusen nor pigmentary changes noted on CFP, and no drusen nor retinal layer disruption on OCT. The exclusion criteria included: (a) Significant medial opacity affecting the quality of multimodal imaging, (b) Presence of significant ocular pathology other than AMD including epiretinal membranes, diabetic retinopathy, glaucoma, and other conditions that may confound grading and VF measurements.

**Figure 1:**
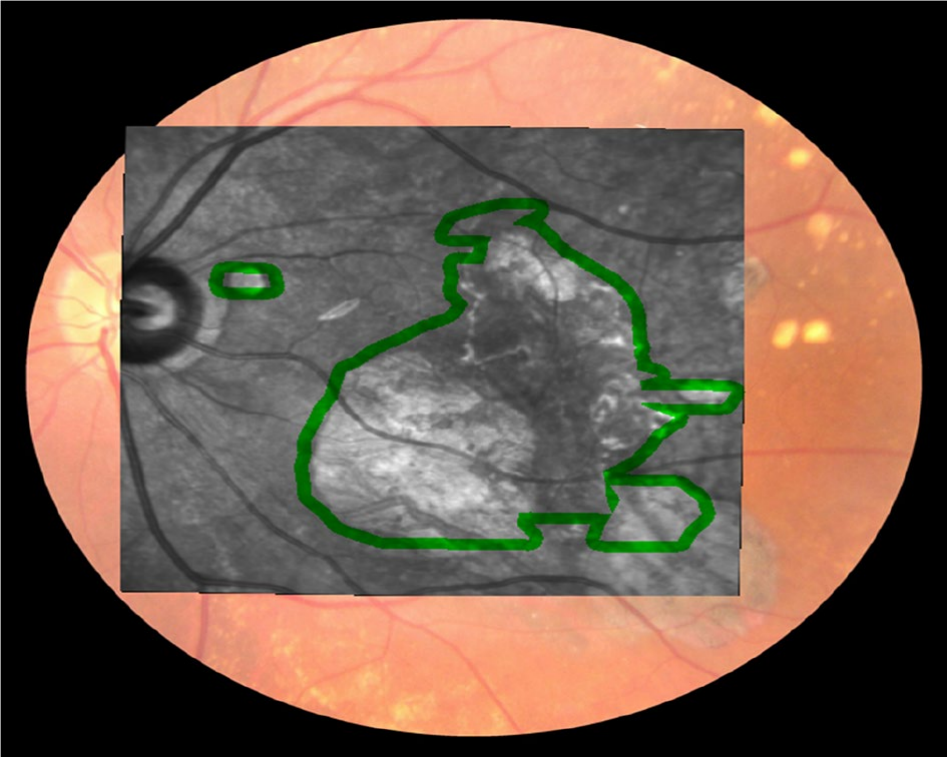
An example of the peri-lesional area of an area of atrophy. The peri-lesional area is represented by the green outline which corresponded to 1 degree of visual angle from the boundaries of the area of atrophy.

For subgroup analyses, AMD eyes were divided into early and intermediate; and advanced AMD (definitions elaborated below). Some patients had AMD changes only in 1 eye, where the fellow eye did not have any changes associated with AMD, i.e. no drusen nor pigmentary changes were noted on CFP, and no drusen nor retinal layer disruption on OCT. These 'fellow eyes’ were analyzed as well. Peri-lesional areas referred to 1 degree of visual angle (0.3 mm border thickness) from boundaries of pathological lesions (Fig. 1). Structurally normal areas referred to areas of the retina in AMD eyes with no apparent pathological lesions, i.e. excluding lesion and peri-lesional areas.

### Study assessment and clinical examination

An experienced retinal specialist performed a dilated fundal examination to confirm the diagnosis of AMD, and verified the absence of ophthalmic pathology in normal controls. Best-corrected VA was assessed with the Snellen chart, and converted to logarithm of minimal angle of resolution (LogMAR) equivalent. Participants then underwent multimodal imaging, with cross-sectional and enface spectral-domain OCT (SD-OCT, Spectralis HRA-2, Heidelberg Engineering, Inc, Heidelberg, Germany), OCTA (OCTA, Angioplex, Cirrus OCT 5000, Zeiss, Dublin, CA, USA), and IR fundus. This involved volume scans capturing images at enhanced depth and high speed over a 6 × 6 mm macular region centered on the fovea, with 25 B-scans (~ 240 µm between each scan).

### Grading of AMD-related pathological lesions

AMD grading was based on the Beckmann Classification for AMD^19^. Advanced AMD refers to the presence of geographic atrophy (GA) and/or neovascular AMD. Early and intermediate AMD refer to the presence of drusens (≥ 63 um in diameter) and/or retinal pigment epithelium (RPE) pigmentary abnormalities, and not meeting the criteria of advanced AMD.

OCT/OCTA images were graded by 2 independent graders, masked to the MP results, with adjudication from a senior retinal specialist. Retinal structures and AMD-related lesions were identified based on the published consensus definitions on terminologies^13^. The RPE was identified as the hyperreflective band between the choriocapillaris and interdigitation zone. The ellipsoid zone (EZ) was identified as the adjacent hyperreflective band above the RPE. The external limiting membrane was identified as the hyperreflective band between the outer nuclear layer and EZ. Pathological lesions were classified into early and acute lesions, including SRF, defined as separation of the neurosensory retina from the RPE by fluid; and IRF, defined as hyporeflective cystoid spaces within the retina. Drusen and PED were categorized together, defined as elevation of the RPE from the underlying Bruch’s membrane. NV was defined as the visualization of a neovascular network on OCTA based on the best fit manual segmentation of the neovascular lesion identified. Late and chronic lesions included fibrosis, defined as an accumulation of well-demarcated hyperreflective material in the subretinal or sub-RPE space that may have a multilaminar appearance; and atrophy defined as the loss of RPE band with associated choroidal hypertransmission.

### Visual function assessed on Microperimetry

Nidek MP-3 (Nidek Co. Ltd, Gamagori, Japan) was performed for all eyes. It enabled automatic eye-tracking with fast acquisition speed, allowing the same retinal foci to be stimulated, increasing accuracy and reproducibility of results. For each eye, a reference enface MP-CFP was first captured. Subsequently, retinal sensitivity points (RSP) were mapped onto MP-CFP based on the standardized protocol for AMD macula analysis, with similar settings to previous studies^20,21^. This comprised a 68-stimuli grid over the central 10° of the macula, 1° diameter yellow circle as the fixation target, standard Goldmann III stimulus with a maximum luminance of 10000 asb. Additional VF parameters measured included fixation area and stability, and distance of fixation point from the fovea.

### Image processing and analysis

**Figure 2:**
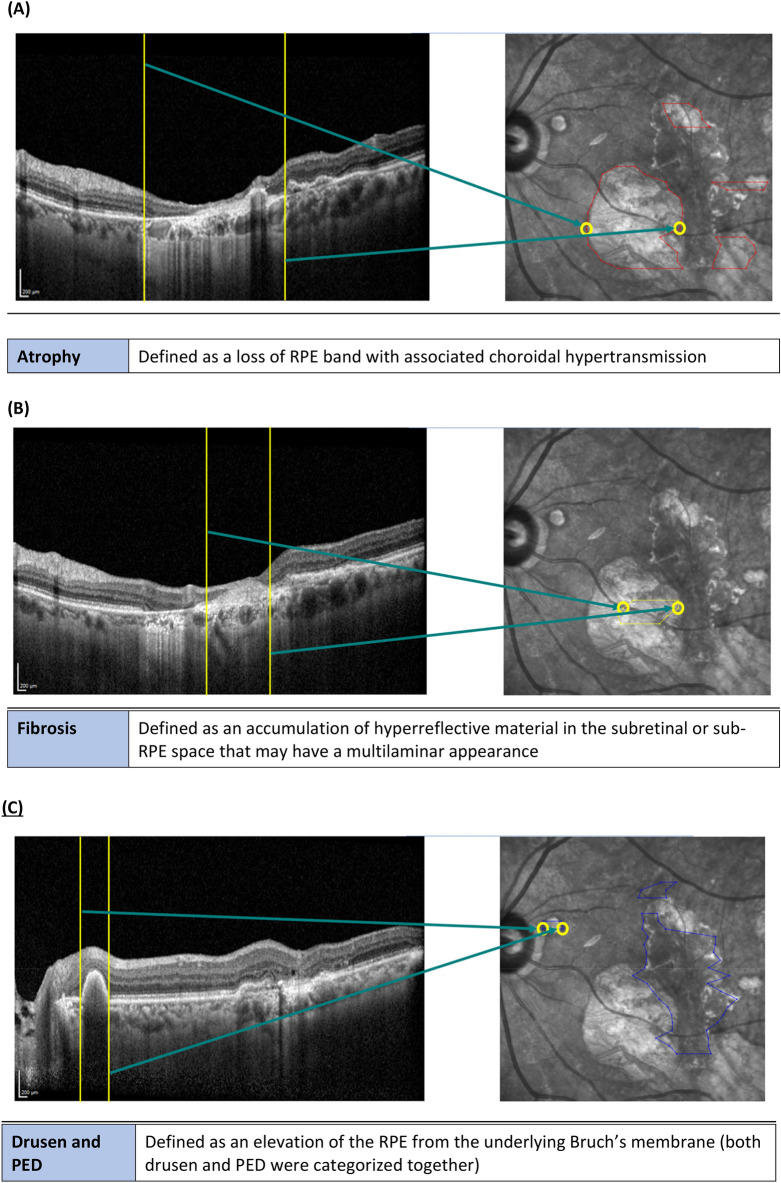

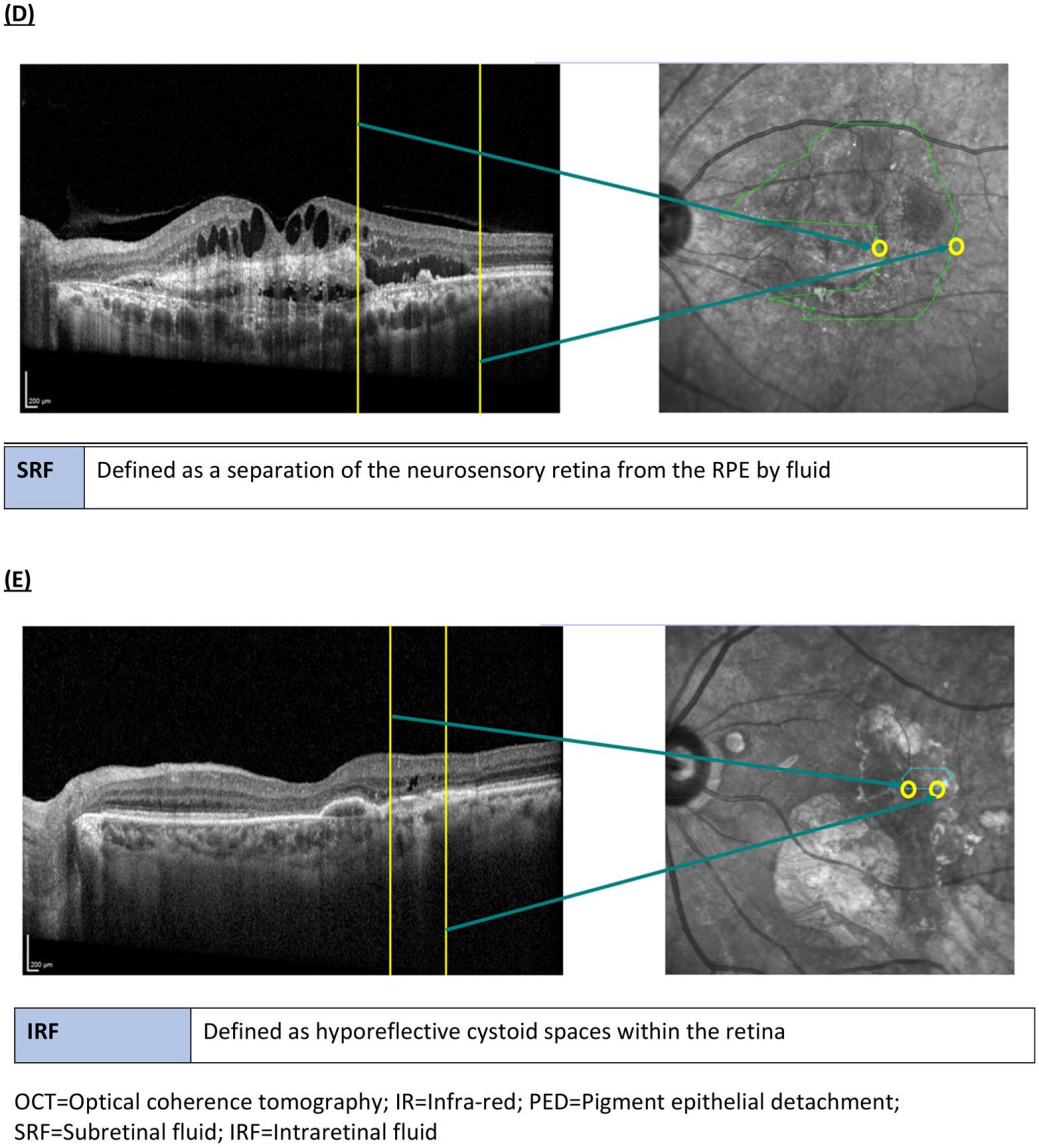
Grading and processing of AMD-related pathological lesions on OCT and IR fundus images. Manual annotation of the coordinates corresponding to boundaries of AMD-related pathological lesions identified on OCT. Using our custom marking tool, these coordinates were translated onto corresponding enface IR fundus images: (**A**) Atrophy, (**B**) Fibrosis, (**C**) Drusen and pigment epithelial detachment, (**D**) Subretinal fluid, (**E**) Intraretinal fluid. The definitions of AMD-related pathological lesions were based on consensus nomenclature by the Consensus on Neovascular Age-Related Macular Degeneration Nomenclature Study Group^44^.

**Figure 3:**
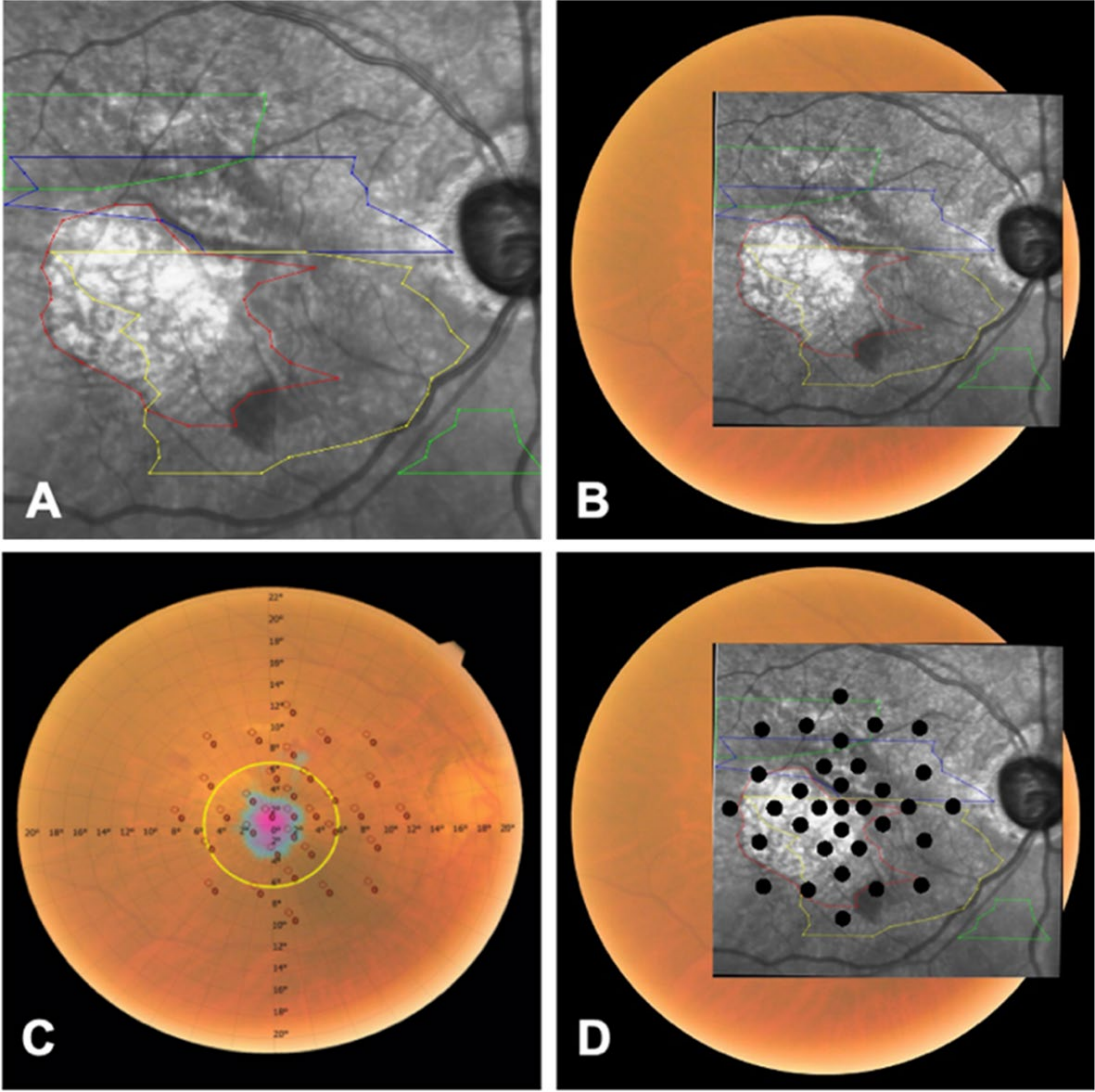
Multimodal imaging overlay. (**A**) Enface IR fundus image with specific pathological lesion masks, that were translated from manual annotation of optical coherence tomography, using our custom marking tool (as shown in Fig. 1) (**B**) Lesion-annotated enface IR fundus image and MP-CFP were superimposed together (**C**) Retinal sensitivity points on MP-CFP at pre-determined protocolized points (**D**) Multimodal imaging overlay enabled topographical correlation of lesion masks on enface IR fundus image and RSP on MP-CFP. *IR* Infra-red, *MP* microperimetry, *CFP* color fundus photograph, *RSP* retinal sensitivity points.

OCT was pre-overlayed onto corresponding IR fundus images within Heidelberg SD-OCT. We designed a custom marking tool using MATLAB, that translated the coordinates of manual annotation of pathological lesion boundaries identified on OCT, onto IR fundus images (Figs. 2A–E and 3A). Manual annotation of the boundaries of NV was performed on enface OCTA. As such, specific lesion masks for each AMD eye were generated. RSP, each represented by a circular mask, were mapped onto MP-CFP at the Nidek MP-3 protocolized points (Fig. 3C).

Next, retinal vasculature masks of lesion-annotated IR fundus images and enface OCTA, as well as MP-CFP were extracted using a series of image processing filters, and superimposed iteratively to obtain the best match (Fig. 3B). The resulting multimodal imaging overlay enabled direct topographical correlation of RSP lying within boundaries of specific lesion masks (Fig. 3D). A 10% area of intersection was taken to determine the corresponding RSP to each lesion mask. All RSP corresponding to each lesion mask were then averaged to obtain the mean retinal sensitivity (MRS) for that lesion.

### Statistical analysis

Statistical analyses, performed on MATLAB, were first conducted to investigate the relationship between AMD and control groups (Tables 1 and 2). Unpaired t-tests were performed for continuous variables and Fischer’s exact test for categorical variables. A resulting p-value ≤ 0.05 was taken as statistically significant. Next, multilevel mixed-effect logistic regression was used to assess the association between various lesion types and controls in terms of MRS. 2 eyes (Level 1) were nested within each subject (Level 2). Age and gender were level 2 predictors; lesion parameters (presence of each lesion type) were level 1 predictors. Univariate analysis of each lesion parameter adjusted for level 2 predictors was first performed, followed by a multivariate analysis of each lesion parameter adjusted for both level 1 and 2 predictors. In the multivariate analysis, parameter adjustments were accounted for where appropriate. Analysis outcomes were presented as odds ratio (exponential of beta-coefficient) and p-values were further visualized in a forest plot.

**Table 1:** Comparison of visual function outcomes between eyes with AMD and normal controls.

By eye	Whole cohort N = 228	All AMD N = 140	P-value* (when compared to controls)	AREDS 1 to 3 N = 30	P-value* (when compared to controls)	AREDS 4 N = 110	P-value* (when compared to controls)	Control N = 66	P-value* (when comparing between AREDS 1 to 3 and AREDS 4 subgroups)
Mean retinal sensitivity dB (SD)	22.3(7.6)	19.2(7.6)	< 0.01	23.1(5.5)	< 0.01	18.1(7.8)	< 0.01	27.8(4.3)	< 0.01
Fixation unstable (%)	127(55.7)	88(62.9)	0.02	18(60)	0.19	70(63.6)	0.01	29(43.9)	0.39
Fixation area mm^2^ (SD)	3.3(2.6)	3.8(2.9)	< 0.01	3.4(2.2)	0.07	3.9(3.0)	< 0.01	2.6(1.9)	0.13
Fixation distance from fovea mm (SD)	0.6(0.6)	0.7(0.7)	< 0.01	0.6(0.6)	0.07	0.7(0.8)	< 0.01	0.4(0.3)	0.09
Visual acuity logMAR (SD)	0.4(0.4)	0.6(0.5)	< 0.01	0.4(0.3)	< 0.01	0.6(0.5)	< 0.01	0.2(0.2)	< 0.01

*p-value calculated by unpaired t-test for continuous variables and fisher test for categorical variables.

**Table 2:** Comparison of visual function outcomes between fellow eyes of AMD eyes and normal controls.

By eye	Fellow eyes of all AMD^ N = 22	P-value* (when comparing to controls)	Fellow eyes of AREDS 1 to 3 N = 4	P-value* (when comparing to controls)	Fellow eyes of AREDS 4 N = 18	P-value* (when comparing to controls)	Control N = 66	P-value* (when comparing between AREDS 1 to 3 and AREDS 4 subgroups)
Mean retinal sensitivity dB (SD)	25.7(3.0)	0.04	27.0(1.4)	0.72	25.4(3.2)	0.03	27.8(4.3)	0.34
Fixation unstable (%)	10(45.5)	1.00	2(50)	1.00	8(44.4)	1.00	29(43.9)	1.00
Fixation area mm^2^ (SD)	2.6(1.6)	0.96	1.9(1.0)	0.46	2.8(1.7)	0.71	2.6(1.9)	0.33
Fixation distance from fovea mm (SD)	0.4(0.3)	0.97	1.0(0.3)	0.60	0.4(0.3)	0.79	0.4(0.3)	0.60
Visual acuity logMAR (SD)	0.2(0.2)	0.73	0.2(0.1)	0.89	0.2(0.2)	0.66	0.2(0.2)	0.77

*p-value calculated by unpaired t-test for continuous variables and fisher test for categorical variables.

^^^Fellow eyes of AMD eyes referred to the other eye of patients with AMD changes in one eye. These fellow eyes did not show any lesions associated with AMD, i.e. no drusen nor pigmentary changes were noted on CFP, and no drusen nor retinal layer disruption on OCT.

## Results

### *Study population* and demographics

**Figure 4:**
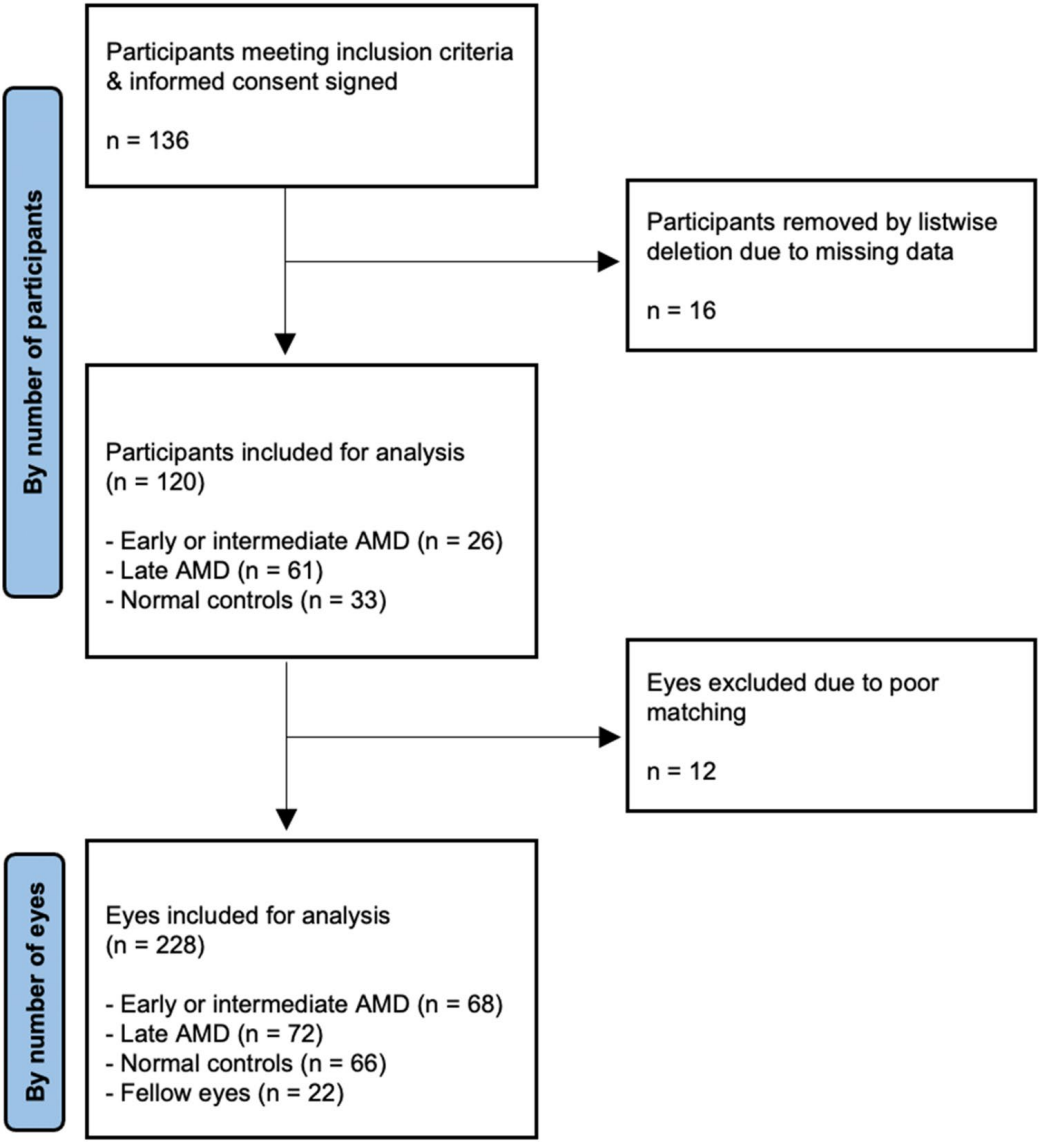
Flow diagram for study population included for analysis.

136 participants were initially included. 16 participants had missing data (e.g. imaging modalities not performed) and were omitted by listwise deletion. The remaining 120 participants included for analysis comprised 27 patients with early/intermediate AMD, 60 patients with advanced AMD, and 33 normal controls. Out of the 240 eyes of these 120 participants, 12 eyes were excluded due to vasculature markings that were not prominent enough for multi-scale template matching. Out of the remaining 228 eyes, 140 were AMD and 66 eyes were controls. Out of the AMD eyes, 30 eyes were early/intermediate AMD, while 110 eyes were advanced AMD. Out of advanced AMD eyes, only 4 eyes had pure GA and the rest were neovascular AMD with 48 eyes showing active disease. 22 eyes were fellow eyes of AMD patients (4 fellow eyes of early/intermediate AMD, 18 fellow eyes of late AMD) (Fig. 4).

The mean age of AMD patients (71.5 years; SD = 8.1 years) was higher compared to controls (65.4 years; SD = 7.7 years (p < 0.01)). Gender and race composition were not significantly different between groups (p > 0.05). 50% (SD = 57.5) of AMD patients and 17% (SD = 51.5) of controls were males. 93.1% of AMD patients and 78.8% of controls were Chinese.

### Comparison of visual function in AMD eyes versus controls

VA in logMAR was significantly poorer in AMD eyes compared to controls. This was consistent in subgroup analysis of early/intermediate AMD and advanced AMD, as compared to controls (Table 1). In terms of VF measured on MP compared to controls, AMD eyes had significantly poorer MRS, larger proportion of unstable fixation, larger fixation area, and fixation point further away from the fovea. MRS reduction was significantly worse in both subgroups; while fixation analyses were only significantly reduced in the advanced AMD subgroup (Table 1).

Notably, VA of fellow eyes of all AMD eyes was similar to controls (0.2, SD = 0.2 versus 0.2, SD = 0.2; p = 0.73). However, MRS of these fellow eyes were significantly lower than controls (25.7 dB, SD = 3.0 dB versus 27.8 dB, SD = 4.3 dB; p = 0.04). MRS of fellow eyes of advanced AMD eyes were also significantly lower than controls (25.4 dB, SD = 3.2 dB versus 27.8 dB, SD = 4.3 dB; p = 0.03), while the rest of subgroup and fixation analyses did not reveal any statistical significant differences (Table 2).

### Analysis of structure–function correlation of specific pathological lesions

With the multimodal imaging overlay, VF on MP were correlated to corresponding AMD-related lesions identified on multimodal imaging. MRS of the total lesion area (16.9 dB, SD = 8.4 dB) in AMD eyes was lower than the MRS of surrounding structurally normal areas (22.2 dB, SD = 6.9 dB) (p < 0.01). Notably, the peri-lesional areas (20.2 dB, SD = 7.6 dB) (Fig. 4) as well as surrounding structurally normal areas (22.2 dB, SD = 6.9 dB) still had lower MRS when compared to controls (27.8 dB, SD = 4.3 dB) (p < 0.01)) (Table 3).

**Table 3:** Mean retinal sensitivities of various pathological AMD lesions.

	Number of eyes	Number of eyes ^#^	Mean area mm^2^ (SD)	Mean number of sensitivity points	Mean retinal sensitivity dB (SD)
Total lesion area	140	134	14.6(11.6)	16.4	16.9(8.4)
Peri-lesional areas ^@^	140	140	4.4(2.3)	6.6	20.2(7.6)
Structurally normal areas^§^^3^	162	160	130.6(11.9)	17.5	22.2(7.0)
Pathological structures					
Atrophy	31	27	4.4(4.4)	5.9	6.2(7.0)
Fibrosis	34	33	5.3(7.5)	6.9	5.5(5.4)
Drusen and PED	131	126	12.4(10.8)	14.4	17.7(8.0)
SRF	33	31	5.4(5.5)	7.0	12.9(7.7)
IRF	26	23	3.3(3.4)	6.0	7.7(7.2)
NV	46	46	8.5(5.2)	11.6	13.2(8.7)
Early and acute lesions (SRF, IRF, PED, NV)	140	127	12.8(10.9)	15.4	17.9(7.9)
Late and chronic lesions (Fibrosis, atrophy)	51	49	6.0(7.6)	7.5	6.4(6.2)

*AMD* age related macular degeneration, *PED* pigment epithelial detachment, *SRF* subretinal fluid, *IRF* Intraretinal fluid, *NV* neovascularization, *MP* microperimetry, *RSP* retinal sensitivity points.

^#^Our multimodal imaging overlay algorithm only took into account MP RSP that intersected lesion masks by at least 10%. Hence, there are some eyes with lesion areas that had insufficient or no areas of intersection with MP RSP, thus did not yield any data for analysis. One example would be eyes with only drusens and PED which are too small in size.

^@^Peri-lesional areas referred to 1 degree of visual angle (0.3 mm border thickness) from boundaries of AMD-related pathological lesions (Fig. 1).

^§^Structurally normal areas referred to areas of the retina in AMD eyes with no apparent pathological lesions, i.e. excluding lesion areas and peri-lesional areas.

**Figure 5:**
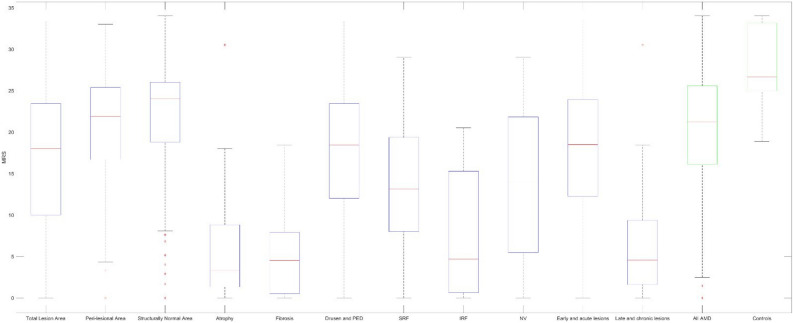
Box-plot diagram of mean retinal sensitivity of various pathological AMD lesions. Peri-lesional areas referred to 1 degree of visual angle (0.3 mm border thickness) from boundaries of AMD-related pathological lesions (Fig. 1). Structurally normal areas referred to areas of the retina in AMD eyes with no apparent pathological lesions, i.e. excluding lesion areas and peri-lesional areas.

The pointwise MRS analysis corresponding to specific AMD-related lesions on multimodal imaging showed: (1) fibrosis (5.5 dB, SD = 5.4 dB), (2) atrophy (6.2 dB, SD = 7.0 dB), (3) IRF (7.7 dB, SD = 7.2 dB), (4) SRF (12.9 dB, SD = 7.7 dB), (5) NV (13.2 dB, SD = 8.7 dB), and (6) drusen and PED (17.7 dB, SD = 8.0 dB). Early and acute lesions grouped together, such as SRF, IRF, PED, and NV, had higher MRS of 17.9 dB (SD = 7.9 dB), as compared to late and chronic lesions, such as fibrosis and atrophy, with largely reduced MRS 6.4 dB (SD = 6.2 dB) (Table 3; Fig. 5). 5.29% (n = 12) of AMD eyes had blood noted on CFP.

At the fovea, 32 eyes with advanced AMD had SRF or IRF. Of all AMD eyes, 93 eyes (66.4%) had drusen or PED; 11 eyes (7.9%) had SRF, 8 eyes (5.7%) had IRF, 11 eyes (7.9%) had atrophy, 13 eyes (9.3%) ad fibrosis, and 35 eyes (25%) had NV.

**Figure 6:**
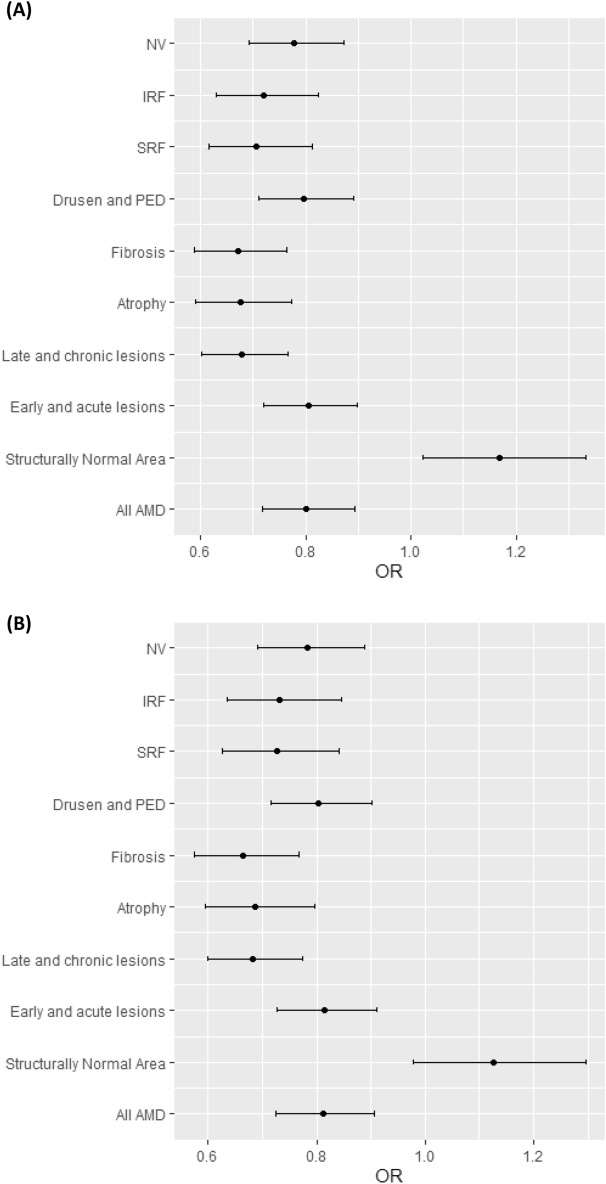
(**A**) Univariate and (**B**) Multivariate forest plots of mean retinal sensitivity of various pathological AMD-related lesions. Late and chronic lesions included fibrosis and atrophy; while early and acute lesions included SRF, IRF, drusen and PED, and neovascularization. Structurally normal areas referred to areas of the retina in AMD eyes with no apparent pathological lesions, i.e. excluding lesion areas and peri-lesional areas. *AMD* age related macular degeneration, *NV* neovascularization, *IRF* intraretinal fluid, *SRF* subretinal fluid, *PED* pigment epithelial detachment, *OR* odds ratio.

Multivariate analysis, adjusted for age and gender, showed that all pathological lesions were significantly associated with reduced MRS (Table 4; Fig. 6).

**Table 4:** Unadjusted and adjusted odds ratio of mean retinal sensitivities of pathological AMD-lesions compared to normal controls.

	Univariable			Multivariable		
	Odds ratio^å^	95% CI	P-value*	Odds ratio^å^	95% CI	P-value*
Total lesion area	0.80	0.72,0.89	< 0.01	0.81	0.73,0.91	< 0.01
Structurally normal areas^§^	1.17	1.02,1.33	0.02	1.13	0.98,1.30	0.10
Atrophy	0.68	0.59,0.77	< 0.01	0.69	0.59,0.80	< 0.01
Fibrosis	0.67	0.59,0.77	< 0.01	0.66	0.57,0.77	< 0.01
Drusen and PED	0.80	0.71,0.89	< 0.01	0.80	0.72,0.90	< 0.01
SRF	0.71	0.61,0.81	< 0.01	0.73	0.63,0.84	< 0.01
IRF	0.72	0.63,0.82	< 0.01	0.73	0.64,0.84	< 0.01
NV	0.78	0.69,0.87	< 0.01	0.78	0.69,0.89	< 0.01
Early acute lesions (SRF, IRF, PED, NV)	0.80	0.72,0.90	< 0.01	0.81	0.73,0.91	< 0.01
Late chronic lesions (fibrosis, atrophy)	0.68	0.60,0.77	< 0.01	0.68	0.60,0.78	< 0.01

*PED* pigment epithelial detachment, *SRF* subretinal fluid, *IRF* intraretinal fluid, *NV* neovascularization.

*P-value calculated by unpaired t-test.

^§^Structurally normal areas referred to areas of the retina in AMD eyes with no apparent pathological lesions, i.e. excluding lesion areas and peri-lesional areas.

^å^Odds ratio calculated as exponential of coefficients.

## Discussion

In summary, as expected, we found that AMD eyes had significantly worse VF compared to controls, as evidenced by decreased VA and MRS, larger proportion with unstable fixation, increased fixation area, and fixation point that was more eccentric and further away from the fovea. This impairment in VF was worse in eyes with advanced AMD versus early/intermediate AMD (Table 1).

More importantly, we demonstrated that in AMD eyes, the reduced MRS was not confined solely to pathological lesion areas. Both peri-lesional and seemingly structurally normal areas of the retina had significantly lower MRS than controls. This suggests that VF may be impaired even when there are no clinically evident structural changes from AMD that can be detected by imaging. Our findings are consistent with previous findings of VF changes in structurally normal regions preceding progression to advanced AMD^22,23^. In particular, we found that fellow eyes of AMD eyes had significantly lower MRS than controls, when reduced VA was not statistically significant. This suggests the potential role of VF assessment using MP, in addition to VA, to detect subtle early changes in areas of structurally normal retina or fellow eyes where there are no clinically evident AMD-related lesions.

Similar studies by Roh et al.^16^ and Sulzbacher et al.^24^ also demonstrated reduced MRS on MP corresponding to AMD-related lesions. MP allows the topographic structure–function correlation by mapping differential RS at specified protocolized point. Its eye-tracking system enhances accuracy and allows fixation analysis, particularly useful in AMD patients with unsteady or extrafoveal fixation. While the former study also compared with controls, it only demonstrated significantly lower MRS in advanced AMD eyes, but was not statistically significant in early/intermediate AMD eyes. In contrast, our study found significant MRS reduction, increasingly unstable fixation, increasingly larger fixation area, and increasing fixation point distance from the fovea, even in early/intermediate AMD with a worsening trend in advanced AMD eyes. Our study also used MP-3, which has a wider light stimulus dynamic range and maximum stimulus luminance of 10,000 asb, as compared to the earlier generation MP-1 (0–20 dB) and Macular Integrity Assessment (MAIA, Center-Vue, Padova, Italy) microperimeters^16,24^.

Another strength of our study lies in the automated custom marking tool and multimodal imaging overlay proposed. This translated specific horizontal coordinates matching lesion boundaries on OCT/OCTA to corresponding IR fundus images, and superimposed onto CFP. As a result, only RSP specifically within lesion boundaries were included, to improve accuracy of lesional MRS measurements. In comparison, Roh et al.^16^ recorded RSP on MAIA corresponding to ETDRS grid sectors where lesions were found. Wu et al.^8^ found no statistical significance in MRS when examining a sector of 2.0 mm^2^ containing areas of atrophy, while their other study^25^, which only looked specifically at MP RSP within atrophic areas, showed significantly reduced MRS. Hence, our custom overlay algorithm enabled a more accurate structure–function correlation of pathological lesions to MP RSP.

In the analysis of AMD-related lesions, we demonstrated that late chronic lesions like fibrosis and atrophy had the lowest MRS. This is consistent with previous studies with extended follow-up showing strong association of fibrosis and atrophy with poorer VA outcomes. However the lesions were classified qualitatively and correlated to VA as the only measure of VF^26,27^. Our study further supports these results by demonstrating that not only is VA reduced, but topographically-mapped MRS within lesion boundaries of atrophy and fibrosis were significantly reduced.

The differentiation of various fluid compartments in AMD and their association with VF has gained substantial interest, especially in the context of finetuning treatment algorithms and outcomes. In our study, IRF areas had lower MRS than SRF (7.7 dB, SD = 7.2 dB versus 12.9 dB, SD = 7.7 dB), which is in accordance with studies demonstrating poorer VA outcomes associated with IRF as compared to persistence of SRF, although MRS difference is small (similar adjusted OR 0.73) and a larger sample size is required to confirm these findings^28^.

Even though PED areas had the highest MRS, the corresponding MRS reduction was greater than controls (17.1 dB, SD = 8.0 dB versus 27.8 dB, SD = 4.3 dB; adjusted OR 0.80; p < 0.01). Existing treatment of PED has been controversial with limited evidence for meaningful visual gains in flattening of PED^29,30^. However, in the context of advanced AMD, our study found corresponding MRS reduction in PED areas, suggesting that PED does affect VF and further longitudinal studies are required to assess if PED flattening can limit MRS reduction.

Furthermore, the methodology of structural–functional assessment described in this study can be applied to investigate newer imaging biomarkers that are important in AMD severity and progression. Features on OCT including intraretinal hyperreflective foci, hyporeflective foci within drusenoid lesions, and subretinal drusenoid deposits (SDD) have been found to be significantly associated with development of late AMD, both macular neovascularization and complete RPE and outer retinal atrophy alone^31^. In particular, studies have found association between SDD and defects in dark adaptation and lower sensitivities when tested using microperimetry, particularly if scotopic microperimetry is performed^32^. Though not the focus of this study, our methodology can potentially be used in future experiments to validate these findings.

With the rapid progress of AI-driven applications, DL models have been developed for the prediction of structure–function correlation in AMD^33,34^. Most of these efforts have targeted prediction of VA from OCT images^35,36^. Specific to microperimetry, Seebock et al. developed a deep learning model (ReSensNet) for the prediction of retinal sensitivities from OCT images with AMD^37^. In their study, specific AMD lesions were not delineated for corresponding RS prediction. The authors observed mismatch in over- and underestimation of RS for certain AMD lesions during qualitative inspection of individual cross-sectional images. Therefore, our proposed methodology to overlay and quantify topographic microperimetric MRS reduction corresponding to AMD-related lesions on multimodal imaging, could supplement this analysis. This could serve as a framework for the development of DL models on structure–function correlation to complement measures of visual function, monitor disease progress and treatment response.

Our study limitations included the cross-sectional design, hence no conclusions on longitudinal changes could be drawn to predict AMD structure–function progression. We also acknowledge that eyes with advanced AMD are heterogeneous. 4 out of advanced AMD eyes had pure GA, which were too few for further analysis. Our study focused on the analysis of topographically-matched MRS of AMD-related lesions and hence, subgroup analysis of different anatomical types^13^ of neovascular AMD was not included and disease activity states were only indirectly identified by the presence of IRF and SRF. Another limitation included the image overlay algorithm that recorded only RSP with ≥ 10% intersection with lesion areas. Hence smaller lesions (e.g. small PED) with insufficient RSP intersection did not yield any data for analysis. Also, image magnification and field-of-view were approximately similar but not specifically standardized. Further, the standardized OCT B-scans being 240 µm apart could have missed small lesions located between successive slices. Nevertheless, our multimodal image overlay enabling detailed topographic MRS assessment within lesion boundaries is novel in AMD structure–function correlation.

## Conclusion

Our study proposed a novel method to accurately quantify topographic MRS reduction corresponding to AMD-related lesions on multimodal imaging, compared to previous studies that only used VA as a VF measure and qualitative grading of pathological lesions. In AMD patients, we demonstrated that peri-lesional, structurally normal areas, and fellow eyes with no gradable AMD lesions still had worse MRS than controls. Hence functional deficits may extend beyond visually apparent structural changes. This detailed topographic structure–function correlation can value-add to VF assessment in prognostication and monitoring of treatment outcomes.

## Data Availability

The data that support the findings of this study may be available on request from the corresponding author.
